# A Multiple Variable-Angle Light Scattering Detector for Gel Permeation Chromatography

**DOI:** 10.6028/jres.096.007

**Published:** 1991

**Authors:** Peter H. Verdier

**Affiliations:** National Institute of Standards and Technology, Gaithersburg, MD 20899

**Keywords:** branched polymers, chain branching, gel permeation chromatography, light scattering, polymer chain branching, polymer molecular weight, size exclusion chromatography, star polymers, star-branched polymers

## Abstract

A light scattering detector has been designed and constructed for use with gel permeation chromatographs. The detector measures light scattered from the eluting sample simultaneously at nine scattering angles, and is connected to a computer-controlled display which exhibits the angular dependence of the scattering in real time while the sample is eluting. Use of the light-scattering detector in conjunction with the usual concentration-sensitive detector allows the direct determination of molecular weight as a function of elution volume, thereby making the chromatographic system “self-calibrating.” Tests of the system with a series of linear and branched polystyrenes suggest that it will be a useful tool for the study and characterization of branched polymers.

## 1. Introduction

Gel permeation chromatography (hereafter GPC; also referred to as size exclusion chromatography, or SEC) is very widely used to estimate molecular weight distribution (MWD) of polymeric materials in solution [1]. However, GPC by itself is merely a chromatographic separation technique, not an absolute method for determining MWD. Therefore, its use for the quantitative estimation of MWD requires either calibrating the chromatographic columns with samples of known molecular weight for each kind of polymer to be studied, or the use of simplifying assumptions, such as the “universal calibration” hypothesis [2], regarding the nature of the chromatographic separation process. However, the addition of a second detector, sensitive to molecular weight, in addition to the usual detector sensitive to concentration, can in principle allow the direct determination of MWD without the need for either calibrants or the aforesaid simplifying assumptions. Detectors have been constructed for this purpose which measure solution viscosity [3] and light scattering [4–10]; some are available commercially. In this paper we report the design, construction and testing of a detector which measures the light scattered over a range of scattering angles by the GPC effluent, The range of scattering angles over which data are taken is adjustable. Extrapolation to zero scattering angle and zero concentration allows determination of the molecular weight of the effluent at each point in the chromatogram, leading to direct determination of the MWD. We demonstrate this “self-calibration” with a series of linear polystyrenes. In addition, we report the results of preliminary studies of a series of star-branched polystyrenes. These studies suggest that this instrument will be a useful tool for the study of branched polymer systems.

## 2. Instrumental Design

**Figure 1:**
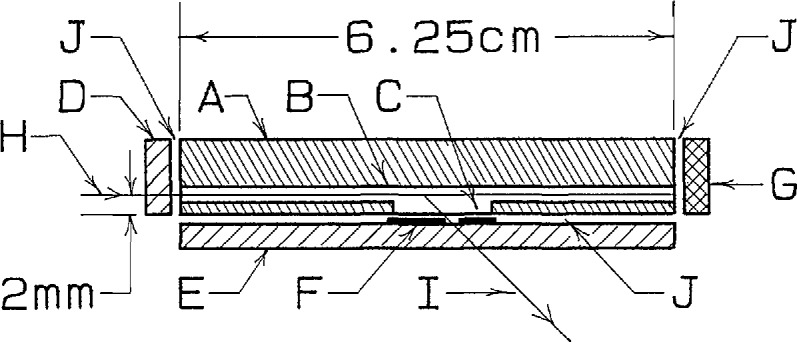
Schematic drawing of multiple-angle flow-through light scattering cell for GPC detector, seen from above and shown in section through the (horizontal) plane of the incident and scattered light paths. A: Cell body (brass); B: Hole, 1 mm dia. (not to scale); C: Milled slot, 1 mm high; D: Entrance window (quartz); E: Exit window (quartz); F: Exit mask with slit (evaporated Cu, thickness not to scale); G: Incident beam dump window (adsorbing glass); H: Incident light path; I: Scattered light path; J: PTFE gaskets (not shown).

The determination of molecular weight by measuring the scattering of light from dilute solution as a function of scattering angle and solution concentration is a well-established technique [11]. In the present application, the requirements of small sample volume for the flowing GPC effluent and the ability to measure scattering simultaneously at several well-defined scattering angles place severe constraints upon the design of the light-scattering detector cell. The design employed in this instrument is shown schematically in figure 1. The cell body is made from a brass block 6.25 cm long. A 1 mm diameter hole is drilled the length of the block, with its center 2 mm from one face as shown in figure 1. Both the incident light and the flowing sample pass along this hole. A milled slot 1 mm wide and 12 mm long meets this hole, allowing a path for scattered light to exit the cell. Incident light enters the cell, and scattered light leaves it, through quartz windows clamped to cover the upstream end of the 1 mm hole and the exit slot, respectively. Unscattered light is removed by a piece of absorbing glass clamped over the downstream end of the 1 mm hole. The two windows and the absorbing glass are sealed by gaskets cut from PTFE film 0.025 mm thick. The part of the exit window which covers the exit slot is covered by an opaque evaporated Cu film except for a slit 2 mm wide, through which scattered light leaves the cell. The inside surfaces of the cell and the evaporated Cu film are blackened chemically with commercial blackening agents [12].^1^ The sample solution enters and leaves the cell through a pair of stainless steel tubes of 0.5 mm i.d. These tubes are silver brazed into holes drilled into the cell body from above at an angle of 30° from vertical, which meet the 1 mm hole just inside the entrance window and exit absorber.

The cell assembly shown schematically in figure 1 is immersed in a paraffin oil bath. The bath vat is made of 100 mm o.d. glass tubing to which a flat glass bottom is fused. A flat face 1 cm wide is ground and polished on the outside of the vat at the entrance of the incident light beam. The cell assembly is positioned in the vat with the exit slit (“F” in fig. 1) centered on the center axis of the vat. Thus the incident light does not travel along a diameter of the vat, but rather travels parallel to and 2 mm away from a diameter. The scattered light is detected by nine photomultiplier tubes positioned on a turret at 15° intervals. The turret can be rotated to vary the 120° range covered by the nine photomultipliers. The angular acceptance and scattering volume are defined by the width of the exit slit, the height of the incident light beam in the sample, and a 3.2 mm diameter circular aperture in front of each detector, at a distance of about 11.5 cm from the exit slit [13].

The incident light beam, of 633 nm wavelength, is obtained from a 5 mW He-Ne laser, and is linearly polarized with the E-vector perpendicular to the plane of the incident and scattered beams (so-called vertical polarization). A pellicle beamsplitter directs a small fraction of the incident light through an absorbing glass filter to a reference photomultiplier for monitoring. A simple biconvex lens of 30 cm focal length reduces the incident beam diameter to about 0.4 mm at the sample cell. The laser, scattering cell vat, and photomultipliers are all enclosed in a light-tight box. An electrically operated shutter at the laser exit window allows detector dark currents to be measured without turning off the laser.

The light-scattering detector cell is inserted in an otherwise conventional GPC system, employing a fixed-volume loop for sample injection upstream of the chromatographic columns, and a detector which senses changes in refractive index (RI). A second, large (2 mL) injection loop between the chromatographic columns and the detectors facilitates calibration measurements as described in the following section. The light-scattering cell is inserted between this injection loop and the RI detector, since the latter is extremely sensitive to even the small back pressure that would be produced by the cell and its connecting tubing if they were connected downstream of it. The stainless steel tubing between the two detectors is kept as short as possible to minimize the volume and consequent time delay between signals from the two detectors. The signals from the scattering and reference photomultipliers, which are operated in current mode rather than photon counting mode, are sent through current-to-voltage converters and amplifiers to a 16-channel, 12 bit A/D converter. Signals from the RI detector, temperature sensor, and sample inject signal are also sent to the A/D converter. Every 100 ms, the 13 signals are digitized by the converter and read by a dedicated minicomputer which processes the input data, writes the results to disk, and sends the scattering data to a video display terminal which plots the angular variation of scattered intensity in real time as the chromatogram is being obtained. Data from runs at several concentrations are then re-read from disk and combined to give molecular weight as a function of elution volume, allowing calculation of the MWD and various average molecular weights.

## 3. Calibration

Before this instrument can be operated as a molecular-weight detector, several calibration measurements must be made. These measurements are necessary to account for the time delays between signals reaching the RI detector and the photomultipliers, to determine the solution injection volume, to establish true scattering angles inside the solution, and to convert scattering signals to Rayleigh ratios. The first two depend only upon the equipment, not upon the solvent used; the last two are solvent-dependent.

### 3.1 Time Delays

Although the volume of the tubing connecting the RI and light scattering detectors has been kept as small as practical, it amounts to about 150 μL, corresponding to a delay between the light-scattering detector and the RI detector of about 10 s at a typical flow rate of 1 mL/min. In addition, the geometry of the sample cell is such that a given part of the solution is “seen” by the nine photomultipliers at slightly different times. At a flow rate of 1 mL/min, the response of the photomultiplier at the largest scattering angle is effectively delayed by about $1\frac{1}{2}$ s relative to the response of the photomultiplier at the smallest scattering angle. The programs which process data from the instrument can readily allow for the relative delays between the different signals, once the delay times are known. The delays are most conveniently determined by injecting a sample between the chromatographic columns and the detectors, and observing the relative times at which the resulting signals reach the RI detectors and the photomultipliers. The time delays, which result from the system volume between the regions “seen” by the various detectors, are proportional to flow rate.

### 3.2 Injection Volume

In order to convert signals from the RI detector to concentrations, which are needed to calculate molecular weights from the scattering data, we need to know the sample injection volume *v*_0_. This volume may be determined by injecting a suitable solution of concentration *c*_0_ and integrating the resulting RI signal *s*(*v*) over elution volume *v*. Since *s*(*v*) is proportional to the concentration *c*(*v*), we set *c*(*v*)*=ks*(*v*), and the total mass *m* of solute injected can be written:

$$m=c_{0}v_{0}=\int cdv=k\int s(v)dv.$$ (1)

The proportionality factor *k* can be determined by injecting a sample of the same material at a known concentration *c*′ (typically much less than *c*_0_) directly into the RI detector, bypassing the chromatographic columns, and observing the resulting signal *s*′. Then the injection volume *v*_0_ is given by:

$$v_{0}=(c^{\prime }/c_{0})\int s(v)dv/s^{\prime }.$$ (2)

### 3.3 Scattering Angles

It will be seen from figure 1 that refraction at the solution-exit window and exit window-bath liquid interfaces will in general cause the scattered light path to be more complex than that shown in figure 1. If the bath liquid is chosen for a close index match with the exit window, then the true scattering angle θ in the solution (measured, as usual, from the direction of the incident light path) is given in terms of the externally measured scattering angle θ_e_ and the indices of refraction *n*_s_ and *n*_b_ of solution and bath by Snell’s law, in the form:

$$n_{s}\cos\text{θ=}n_{b}\cos{\text{θ}}_{e}.$$ (3)

Thus the index of refraction of the solution must be known in order to determine the scattering angles. The difference between θ and θ_e_ is negligible for solutions with indices near *n*_b_, which is about 1.5 for the paraffin oil used as the bath liquid. However, the difference can become large for measurements on samples in solvents such as tetrahydrofuran or water, with indices of 1.4 or less, when θ_e_ is far from π/Z. (For example, for a solution of index 1.4, an external angle θ_e_ of 30° corresponds to a true scattering angle θ of less than 22°.)

### 3.4 Rayleigh Ratios

In order to convert scattering signals to the Rayleigh ratios needed for the calculation of molecular weights, the detector systems must be calibrated against a material of known Rayleigh ratio. As a practical matter, this means determining the Rayleigh ratio of the solvent as a function of scattering angle and using it as a working standard. In principle, this could be done by measuring solvent scattering as a function of scattering angle with a conventional light scattering photometer, relative to a standard of known Rayleigh ratio. In practice, however, with the present scattering cell we have been unable to prevent a certain amount of stray light, scattered from cell windows, cell walls, etc., from reaching the photomultipliers. If the amounts of stray scattering detected are constant in time and not too large, they may be determined and treated as instrumental parameters by measuring the relative scattering signals from solvent and some convenient solution both in the scattering cell and in a conventional light scattering photometer. At each scattering angle θ*_i_*, if ϵ*_i_* is the ratio of stray scattering to solvent scattering, then we have:

$$1+\epsilon_{i}=(r_{t,i}-1)/(r_{a,i}-1),$$ (4)

where *r*_t_*_,i_* is the “true” ratio of solution scattering to solvent scattering at θ*_i_*, measured with the conventional photometer, and *r*_a,_*_i_* is the “apparent” ratio measured with the present instrument. For the work reported here, using toluene as a solvent, the stray scattering turned out to be a few percent of solvent scattering except at the smallest scattering angle employed, where it was 23% of solvent scattering.

## 4. Operation

As with conventional chromatography, when maximum precision is desired, each data collection injection is preceded by a trial injection during which the instrumental gains are set so that the maximum signal from each detector is close to the largest that can be digitized by the A/D converter. For this purpose, the video display terminal attached to the computer serves as a convenient 16-channel output meter, allowing gains in the photomultiplier and RI detector channels to be set as the sample is eluted.

After instrumental gains have been set, a data collection injection may be made. As with any determination of molecular weight by light scattering, dark signals, working standard signals and solvent signals must be obtained and used in conjunction with the signals from the sample solutions. (For the present instrument, as previously noted, the solvent is used as the working standard.) Prior to actual injection, therefore, a data collection run begins by recording the photomultiplier signals with the laser shutter closed. Running averages of the dark signals so obtained are calculated by the computer and displayed upon operator request at the video display terminal, together with sample standard deviations of the individual samples and of their means.

When the dark signals are deemed sufficiently precise, their collection is ended and the final averages and standard deviations are stored. The laser shutter is opened, a few seconds are allowed for the detector channels to settle down, and collection of solvent signals begins. As with the dark signals, running averages and standard deviations are displayed when desired while the data are being collected, to aid in deciding when the solvent/working standard readings are sufficiently precise.

After the collection of solvent/working standard readings is complete, the sample is injected. A switch on the sample injection valve gives the computer a reference point for zero time/elution volume for the chromatogram. When sample elution from the columns begins, the video display terminal plots the angular dependence of the scattering, updated every few seconds, to allow continuous verification that the experiment is under control.

## 5. Data Treatment

As previously described, the scattering and other signals generated by the instrument are sent to a 12 bit A/D converter. Every 100 ms, the minicomputer used for data acquisition reads the converter, stores its readings in memory, and updates an internal clock reading. Before being analyzed, the input data are pre-treated by routines which can be thought of as simulating electronic filters, delay lines, etc. The pre-treated data are then sent to a video display terminal which calculates and displays the angular dependence of scattering in real time, and to a hard disk for later analysis. Finally, after the experimental data have all been obtained, the results are read back from disk, combined and analyzed to obtain molecular weight distribution and other desired quantities.

### 5.1 Pre-Analysis Processing

The A/D converter used in this instrument converts input signals in the range 0 to 5 V to integers in the range 0 to 4095. Input signals less than 0 V or greater than 5 V result in integer values of 0 and 4095, respectively. These values are therefore used as out-of-range indicators. In the data-collecting phases of operation, the input signals are first examined. If any are found equal to either 0 or 4095, the values in all the channels are displayed with an error message and the run is terminated. If not, the input values are compared with running minimum and maximum values for each channel, which are updated as necessary and displayed at the end of each run, as an aid to setting gains for subsequent runs. The input values *I* are then added into weighted running sums, by replacing each sum *S* by *S*′, given by:

$$S^{\prime }=(15S+I)/16,$$ (5)

an operation which has the same effect as a lowpass RC filter with a time constant of 16 input readings, or 1.6 s. Finally, the filtered values are stored in arrays which compensate for the differences in the times at which a given part of the eluting liquid is sensed by each of the detectors. The arrays, one for each photomultiplier and one for the RI detector, are large enough to contain all the readings taken during the maximum time difference between photomultiplier channels, and are filled cyclically. Every 1.6 s a value is extracted from each array, with offsets in the array indices corresponding to the sensing time differences between the photomultipliers. The offset in the array of RI detector values is chosen to make the delay between the extracted RI signal and the photomultiplier signals precisely 9.6 s, or 6 samples.

### 5.2 Pre-Injection Data Treatment

During the collection of dark current data, the photomultiplier signals, pre-processed as previously described, are added into running sums and sums of squares. When requested by the operator, these are used to form mean values, sample standard deviations, and sample standard deviations of the mean, which are then displayed. When the operator ends the dark current collection, the final values of these quantities are calculated, displayed and written to disk for later use analysing the data.

During the collection of solvent/working standard readings, the previously-obtained mean dark currents are subtracted from each of the sampled photomultiplier signals, and the differences are divided by the reference photomultiplier signal less its mean dark current. The resulting ratios are added into running sums and sums of squares. Upon request, these are used to form and display mean values, sample standard deviations, sample standard deviations of the mean, and relative sample standard deviations of the mean. As with the dark currents, when the collection of solvent/working standard readings is finished, the final values are calculated, displayed and written to disk. In addition, the mean values are sent to the video display terminal for use in calculating and displaying the angular dependence of the scattering after sample injection.

### 5.3 Post-Injection Data Treatment

After the switch on the sample injection valve has informed the program that a sample has been injected, data from the photomultipliers are treated in the same way as already described for solvent data collection, except that instead of being added into running sums and sums of squares, they are written to disk every 1.6 s, together with the RI detector data and a sample count which gives elapsed time since injection in units of 1.6 s. In addition, every 1.6 s the smoothed detector signals *S* are further smoothed by adding them into weighted display sums *S*_d_, by replacing each value of *S*_d_ by *S*′_d_, where:

$$S_{d}^{\prime }=(3S_{d}+S)/4,$$ (6)

giving the effect of an additional low-pass RC filter with a time constant of 6.4 s. Every 6.4 s these smoothed signals are sent to the video display terminal, which calculates and displays plots of reciprocals of the differences between solution scattering and solvent scattering, corrected for stray scattering by the use of eq (4), vs sin^2^(θ/2), for scattering angles θ calculated from eq (3). The plots so displayed are used only to provide real-time assurance that sensible data are being obtained; they are not used in the final analysis of the results.

### 5.4 Subsequent, Post-Experiment Treatment

Each chromatogram obtained as described in the preceding sections produces one file of scattering data and RI data as functions of elution volume. At least two such chromatogram files, obtained at different solute concentrations, are needed to obtain molecular weights from the scattering data, to allow extrapolation to the zero-concentration limits. In addition, in order to obtain concentrations from the RI data, baselines must be determined and subtracted from the RI data in each file. The processing needed to convert the files produced during the chromatographic runs into calibration parameters is accomplished by a series of programs after the chromatograms have been run and the raw data files obtained.

#### 5.4.1 Baseline Determination

The stability of the chromatographic RI detector is not sufficient to allow a solvent reading to be obtained by simply averaging before a run, as is done with photomultiplier dark currents and solvent/working standard readings. Instead, the file originally written to disk is read and the RI signal plotted vs. elution volume. Up to three regions of apparent baseline are located by eye on the plot (typically, one each before and after the sample starts to elute, and optionally a third region after the “trash peaks”). The chosen regions are specified to the program, which then determines the baseline, by unweighted linear least squares, as a linear function of elution volume in the chosen regions, adds the calculated baseline to the plot of RI signal, and integrates the RI signal less baseline over a user-specified region. It then copies the portion of the input file that was selected for integration out to disk, preceded by the baseline parameters and the value of the integral, and with the time-lag between the RI detector and photomultiplier readings removed.

#### 5.4.2 Determination of Molecular Parameters

Two or more files produced as just described, containing data at two or more concentrations, are next combined to give molecular parameters as functions of elution volume. At each sampling time, the smoothed scattering signals *S*′ in eq (5) are read in from these files for each scattering angle θ*_i_* and concentration *c_j_.* Let us change notation by replacing *S*′, which was defined in eq (5), by *S*(*x_i_,c_j_*), where *x_i_*=sin^2^(θ*_i_*/2). The solvent signal at θ*_i_* is then just *S*(*x_i_*,0). Form the array

$$y_{ij}=c_{j}K_{i}S(x_{i},0)/[S(x_{i},c_{j})-S(x_{i},0)],$$ (7)

where:

$$c_{j}=g_{j}c_{0}v_{0}/\int g_{j}(v)dv,$$ (8)

*g_j_* is the RI detector signal less baseline,
$\int g_{j}(v)dv$ is its integral over elution volume *v*,
*c*_0_ and *v*_0_ are the concentration and volume of sample injected, and the factor *K_i_* is given by:

$$K_{i}=4{\text{π}}^{2}{\left(dn/dc\right)}^{2}/[{{\text{λ}}_{0}}^{4}N_{A}R_{V}(1+\in_{i})],$$ (9)

where:

d*n*/d*c* is the differential refractive index of the solution,
λ_0_ is the vacuum wavelength of the incident light,
*N*_A_ is Avogadro’s number,
*R*_V_ is the Rayleigh ratio of the solvent/working standard for vertically polarized radiation, and
ϵ*_i_* is the correction for stray scattering at θ*_i_*, determined from eq (4). The values *y_ij_* are then fitted by unweighted linear least squares to the form:

$$y_{ij}=P+Qx_{i}+Rc_{j},$$ (10)

determining *P*, *Q*, and *R*.

Finally the weight-average molecular weight *M*_W_ mean-square <*s*^2^>, radius and second virial coefficient *A*_2_ are obtained as:

$$M_{w}=P^{-1},$$ (11)

$$<s^{2}>=3{\left(4\text{π}n_{s}/\lambda_{0}\right)}^{-2})Q/P,$$ (12)

$$A_{2}=R/2;$$ (13)

these quantities are calculated, written out to disk, and sent to a plotter.

#### 5.4.3 GPC Calibration

The file of molecular weight vs elution volume obtained for a given sample may be used to calibrate the GPC columns for that sample. Its MWD may then be calculated from concentration data vs elution volume. In this sense, the instrument is self-calibrating; only the sample to be analyzed is necessary. However, the precision of the molecular-weight data naturally falls off in the wings of the chromatogram, where sample concentrations are small. If, as is frequently the case, additional samples of the same type of polymer as the sample under study but of different molecular weights are available, calibration data may also be obtained from them. The final calibration of the columns for a given polymer may then utilize data obtained for all the available samples of that polymer, resulting in a more precise calibration than could have been obtained using data from only one sample. The procedure employed here is to fit all the available values of *M*_w_ vs *v* (which may, of course, be only the data for the sample being analyzed) to the form

$$\ln M_{w}=A+Bv$$ (14)

by unweighted linear least squares. The distribution *W*(*M*) in molecular weight *M* is then given in terms of the calibration parameters *A* and *B* and the concentration data *c*(*v*) for a given injection by:

M=exp(A+Bv), (15)

$$W(M)=c(v)/[c_{0}v_{0}B\cdot \exp(A+Bv)],$$ (16)

and the classical average molecular weights *M*_n_, *M*_w_, etc., may be obtained from the moments of *W*(*M*) in the usual way.

## 6. Results

As a test, the instrument was tried out on two series of polystyrenes in toluene at room temperature. The first was a series of linear polystyrenes, the second a series of star-branched polystyrenes. For this preliminary study, only the molecular-weight data were analyzed; mean-square radius and second virial coefficient are subjects for future work.

### 6.1 Linear Polystyrenes

**Figure 2:**
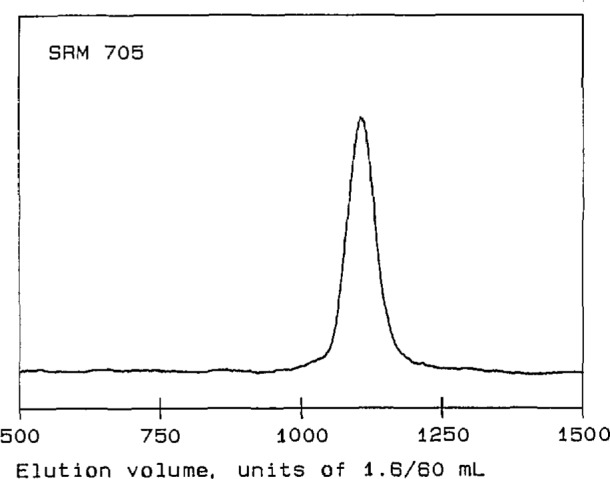
Chromatogram of polystyrene Standard Reference Material 705. The ordinate is a linear function of solution concentration in arbitrary units; the abscissa is elution volume in units of 1.6/60 mL.

Four linear polystyrene Standard Reference Materials (SRMs) [14] were used for initial tests of the performance of the detector. Their molecular-weight characteristics are summarized in table 1. SRMs 1478, 705, and 1479 are anionically polymerized materials, with relatively narrow MWDs and with *M*_w_’s of approximately 4×l0^4^, 2×10^5^, and 1×10^6^, respectively. SRM 706 is a thermally polymerized material, with a relatively broad MWD and an *M*_w_ of about 3×10^5^. The chromatograms of these four linear polystyrenes, shown in figures 2 through 5, are obtained from the RI detector signal [15]. They exhibit the single, narrow peaks expected for the anionically polymerized materials, and a much broader peak for SRM 706.

**Figure 6:**
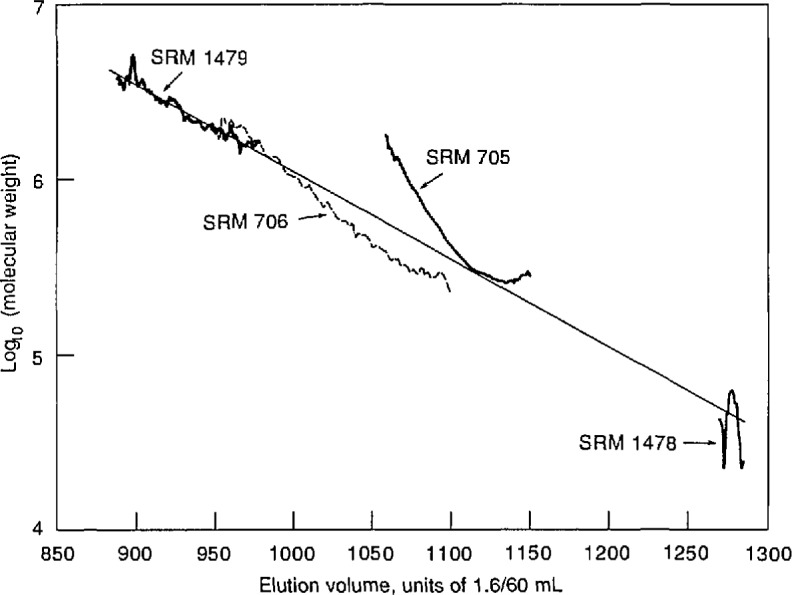
Semilog plots of molecular weights of four linear polystyrenes, SRMs 705, 706,1478, and 1479, vs elution volume in units of 1.6/60 mL. Also shown is the straight line resulting from an unweighted linear least-squares fit of the logarithm of molecular weight to elution volume.

Plots of molecular weight vs elution volume for these linear polystyrenes, obtained from the light scattering detector and RI detector signals as described in the preceding section, are shown in figure 6. To obtain these molecular weights, using eqs (7–11), the Rayleigh ratio *R*_V_ of toluene was taken [16] as 14.0×10^−6^ cm^−1^, a nominal value [17] of 0.11 mL/g was used for the differential refractive index of polystyrene in toluene at a wavelength of 633 nm, and the sample injection loop volume was taken to be the vendor’s nominal value. Also shown in figure 6 is the straight line obtained by fitting the logarithm of molecular weight for all four samples to a linear function of elution volume by un-weighted linear least squares.

It will be seen from figure 6 that the data for SRMs 706, 1478, and 1479 do indeed appear to establish a calibration of the chromatographic columns for linear polystyrene. (The large variation in the molecular weights obtained for SRM 1478 is presumably a consequence of the relatively small scattering strength of this rather low molecular-weight material.) In contrast, the data for this sample of SRM 705 lie almost entirely above the calibration line, touching it only in a short region near the lowest molecular weights for which there are data. However, comparison of figures 2 and 6 shows that the molecular-weight curve for SRM 705 lies close to the calibration line in the region where the peak of the chromatogram occurs. A curve lying above the calibration for linear material would be expected if the sample contains branched components, which have smaller dimensions than linear chains of the same molecular weight, and which therefore leave the chromatographic columns at larger elution volumes. Thus it appears that most of the material in SRM 705 is linear, but there may be small amounts of branched material in the high molecularl-weight tail of its MWD. The presence of star-branched components in SRM 705, due possibly to side reactions having occurred during chain termination, has occasionally been suggested.

**Figure 7:**
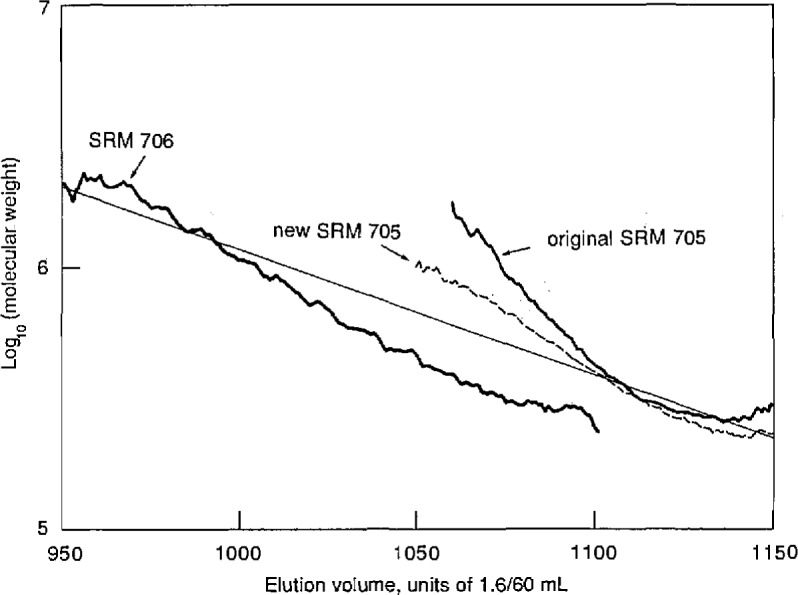
Semilog plots of molecular weights of original and new samples of polystyrene SRM 705, and the original sample of SRM 706, vs elution volume in units of 1.6/60 mL.

The sample of SRM 705 used in the foregoing analysis had been stored in our laboratory for many years, with no special attention to storage conditions. A second series of runs was carried out using another sample of the same material which had been stored in different rooms under slightly different conditions. The results for the two samples are compared in figure 7. It will be seen that they are in close agreement in the elution volume range containing most of the material, but differ in the high molecular-weight tail, where the second sample shows considerably less evidence of branching than does the original sample. Replicate measurements on a single sample reproduced these features. These findings, if confirmed by further studies, suggest that the instrument may well be useful for investigating subtle structural variations in studies of polymer aging and similar phenomena.

Finally, comparison of figure 6 with the data in table 1 shows that the molecular weights obtained for all the samples are consistently higher than the values obtained by conventional methods. The present study was carried out primarily to demonstrate the utility of the instrument for obtaining qualitative information about molecular weight distributions, and the steps needed to obtain absolute accuracy were not taken. In particular, the sample injection volume, which according to eqs (7–11) is inversely proportional to the calculated value of molecular weight, was not determined, but instead was taken equal to the nominal volume of the injection loop. This value could easily be in error by 20%, leading to corresponding errors of the same size in the calculation of molecular weights. However, careful determination of injection volume and the other calibration parameters in future studies should lead to molecular weights as accurate as those obtained by conventional light-scattering methods.

### 6.2 Star-Branched Polystyrenes

**Figure 8:**
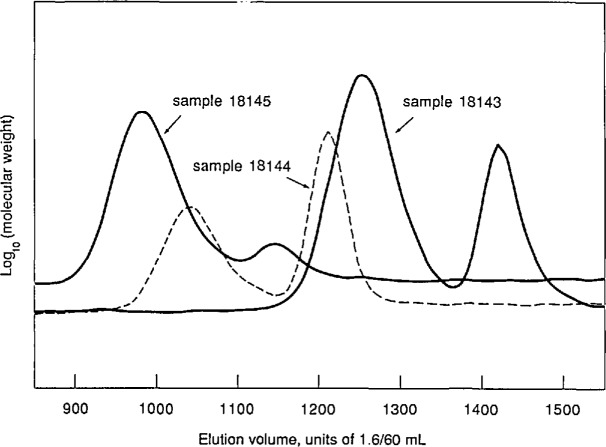
Chromatograms of three star-branched polystyrenes used for tests of the light scattering detector. The ordinate is a linear function of solution concentration in arbitrary units; the abscissa is elution volume in units of 1.6/60 mL.

**Figure 9:**
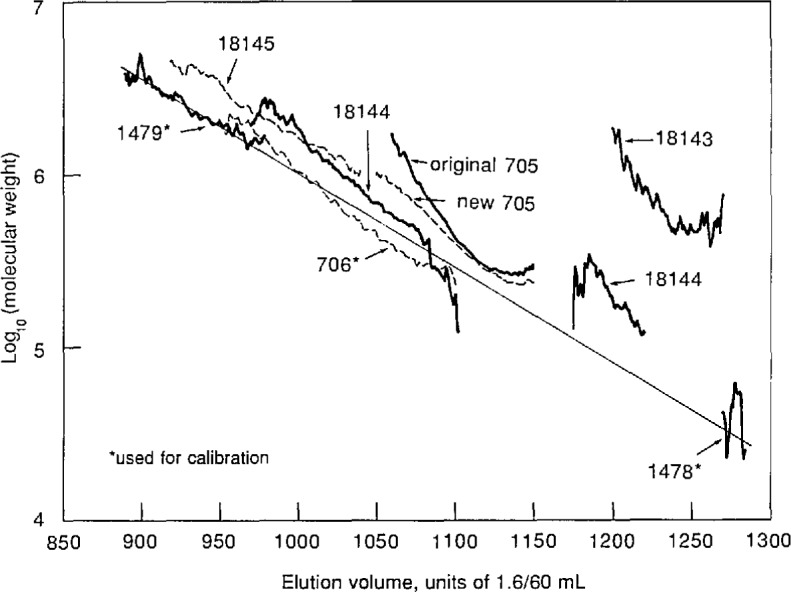
Semilog plots of molecular weights of linear [14] and star-branched [18] polystyrenes vs elution volume in units of 1.6/60 mL. Also shown is the straight line resulting from an unweighted linear least-squares fit of the logarithm of molecular weight to elution volume, using just the three linear polystyrenes marked with asterisks.

Three commercially available star-branched polystyrenes were studied. These materials, obtained from Polysciences, Inc. [18], are listed by the vendor as “Star Shaped Polystyrene”; their stated properties are shown in table 2. Their chromatograms are shown in figure 8; all three are bimodal. Figure 9 is a replotting of the molecular weight vs elution volume data shown in figures 6 and 7, with the results for the star-shaped polystyrenes added. The straight line also shown in figure 9 is the calibration obtained using linear polystyrene samples 706, 1478, and 1479. (Sample 705 was omitted from this calibration because of doubts about its linearity, as previously discussed.) For star-branched samples 18143 and 18145, sufficiently large scattering signals for molecular-weight calculations could only be obtained for the components corresponding to the lower elution-volume peaks. For star-branched sample 18144, however, molecular-weight data were obtained for both components, as shown in figure 9.

Table 3 summarizes the molecular-weight information at chromatographic peaks given in figures 2 through 5, 8, and 9. Shown are values *M*_GPC_ of the *apparent* molecular weight inferred from the elution volume at the peak and the calibration curve for *linear* polystyrene, together with the values *M*_LS_ obtained from the light-scattering data. Also shown are values of the ratio *M*_LS_*/M*_GPC_, whose deviation from unity depends upon branching.

A plausible interpretation of the bimodal chromatograms obtained for the three star-branched samples might be that in each case, the peak at higher elution volume arises from unreacted linear “arm” molecules and the peak at lower elution volume arises from star molecules. Comparison of the values of “Arm *M*_n_” in table 2 with the values of *M*_GPC_ in table 3 shows that the data are reasonably consistent with this supposition. However, for sample 18144, for which light-scattering data were obtained for both peaks, it is clear from figure 9 and table 3 that the higher elution-volume component of sample 18144 is not linear polystyrene. In fact, the molecular-weight values for this peak are perceptibly farther above the linear calibration curve than are the molecular-weight values for the lower elution-volume component of this sample. The molecular weights obtained for the lower elution-volume components of all three star-branched samples lie above the curve for linear molecules, as expected, and the data for the lower elution-volume component of sample 18143 lie farther above the linear curve than do those for the other two samples, consistent with this sample being more highly branched than the others, with 12 arms to their 6.

If we assume that the components of the star-branched samples shown in figure 9 are indeed equal-arm star molecules, we can attempt rough estimates of the numbers of arms for these materials. The most readily accessible measure of branching in our data is the ratio *M*_LS_/*M*_GPC_, which is the ratio of the molecular weight of the molecule of interest to the molecular weight of a *linear* molecule eluting from the chromatographic columns at the same time. Assuming universal calibration, we can write:

$$M_{\text{LS}}{\left[\text{η}\right]}_{\text{LS}}=M_{\text{GPC}}{\left[\text{η}\right]}_{\text{GPC}},$$ (17)

where [η]_LS_ and [η]_GPC_ are intrinsic viscosities corresponding to *M*_LS_ and *M*_GPC_, respectively. The intrinsic viscosity [η]_LS_ is related to the intrinsic viscosity [η]′ of a linear chain of molecular weight *M*_LS_ by the viscosity branching index *g*′, given by

$$g\prime ={\left[\eta \right]}_{\text{LS}}/[\eta ]\prime ,$$ (18)

and [η]_GPC_ and [η]′, which are both intrinsic viscosities of linear chains, are given in terms of empirical parameters *K* and α by the Mark-Houwink relation:

$${\left[\text{η}\right]}_{\text{GPC}}=K{\left(M_{\text{GPC}}\right)}^{\text{α}},$$ (19)

$$[\text{η}]\prime =K{\left(M_{\text{LS}}\right)}^{\text{α}},$$ (20)

Substituting eqs (18–20) into (17), we obtain:

$$M_{\text{LS}}/M_{\text{GPC}}={\left(g\prime \right)}^{-1/(1+\text{α})}.$$ (21)

For star-branched polystyrenes in good solvents, Bauer et al. [19] have given ratios *R*_V_*/(R*_V_)_a_ of equivalent radii *R*_V_ of star-branched molecules to the equivalent radii (*R*_V_)a of their component arms, related to the values of *g*′ by:

$$g\prime ={\left[R_{V}/{\left(R_{V}\right)}_{a}\right]}^{3}f^{-(1+\text{α})},$$ (22)

for stars of *f* arms, giving finally

$$M_{\text{LS}}/M_{\text{GPC}}=f{\left[R_{V}/{\left(R_{V}\right)}_{a}\right]}^{-3/(1+\alpha )}.$$ (23)

In order to make use of eq (23), we need to know α for polystyrene in toluene at room temperature. A review by Wagner [20] lists four values of α for polystyrene in toluene at 25 °C ranging from 0.71 to 0.75, with a mean of 0.73. Using this value, eq (23) becomes:

$$\begin{array}{r} M_{\text{LS}}/M_{\text{GPC}}=f{\left[R_{V}/{\left(R_{V}\right)}_{a}\right]}^{-3/1.73}\\ =f{\left[R_{V}/{\left(R_{V}\right)}_{a}\right]}^{-1.73}. \end{array}$$ (24)

For *f* = 6 and *f* = 12, Bauer et al. [19] give values of 2.33 and 2.93, respectively, for *R*_V_/(*R*_V_)_a_, giving values of 1.4 and 1.9 for the ratios *M*_LS_/*M*_GPC_. The ratios obtained for the lower elution-volume components of star-branched samples 18144 and 18145 are seen to be roughly consistent with the calculated value of 1.4 for 6-armed stars. However, the higher elution-volume component of sample 18144 has a value close to that calculated for a 12-arm star, rather than the value of unity for a linear chain. Bauer et al. do not give ratios for polystyrene stars for values of *f* greater than 12. However, for polyisoprene stars, which have values of *R*_V_/(*R*_V_)_a_ almost identical with those for polystyrene stars at the *f*-values for which data for both are available, they report values of *R*_V_/(*R*_V_)_a_ for *f*-values up to 56.2, for which they obtain *R*_V_/(*R*_V_)_a_ = 4.50. At this *f*-value, eq (23) gives a value of only 4.1 for *M*_LS_*/M*_GPC_, nowhere near the value of 12 found for the lower elution-volume component of sample 18143. Thus this component appears to have a structure considerably more compact than that of a simple equal-armed star.

## 7. Conclusion

Despite the crudeness of these preliminary results, it seems clear that the multiple-angle light scattering detector can be a powerful tool for the study of branched systems. Used in conjunction with other detectors in gel permeation chromatography, it should also serve as a valuable aid in the study of copolymer and other complex systems. Improvements in instrumental sensitivity will also allow direct determination of molecular size and interactions in solution via eqs (12) and (13).

## Figures and Tables

**Figure 3:**
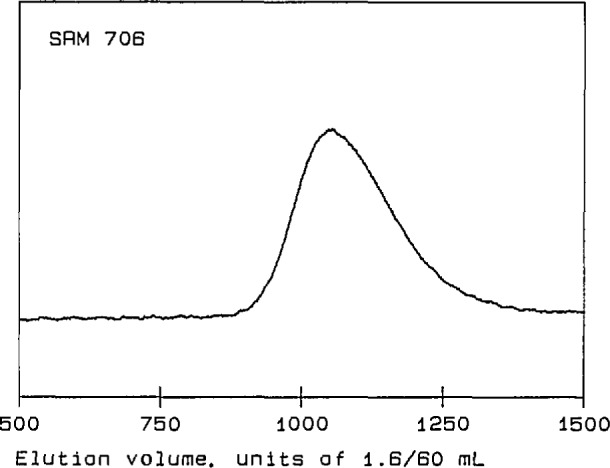
Chromatogram of polystyrene Standard Reference Material 706. The ordinate is a linear function of solution concentration in arbitrary units; the abscissa is elution volume in units of 1.6/60 mL.

**Figure 4:**
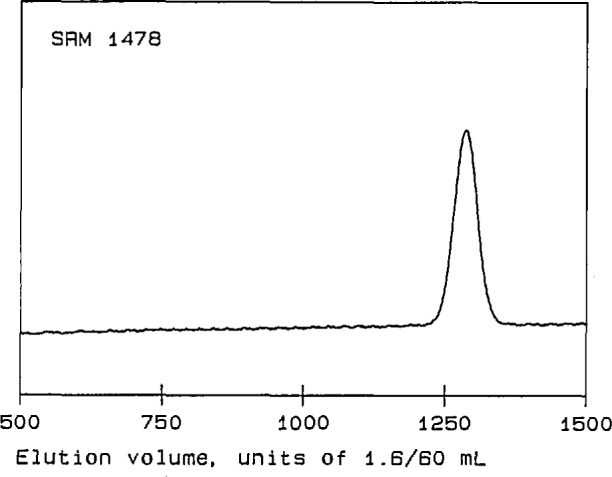
Chromatogram of polystyrene Standard Reference Material 1478. The ordinate is a linear function of solution concentration in arbitrary units; the abscissa is elution volume in units of 1.6/60 mL.

**Figure 5:**
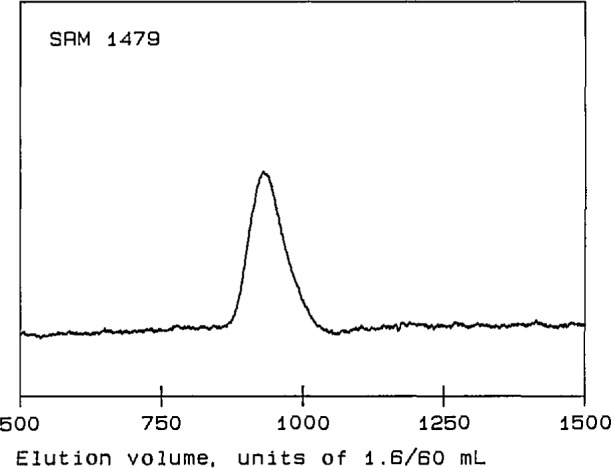
Chromatogram of polystyrene Standard Reference Material 1479. The ordinate is a linear function of solution concentration in arbitraty units; the abscissa is elution volume in units of 1.6/60 mL.

**Table 1:** Linear polystyrenes used for tests of the light scattering detector

No.^a^	Width of MWD	Weight-average molecular weight, *M*_w_, g/mol
705	Narrow	2×10^5^
706	Broad	3×10^5^
1478	Narrow	4×10^4^
1479	Narrow	1×10^6^

a Standard Reference Material No. [14].

**Table 2:** Vendor-supplied characteristics of star-branched polystyrenes used for tests of the light scattering detector

No.^a^	Molecular weight	Arm *M*_n_	No. of arms
18143	90,200	7,000	12
18144	367,300	59,200	6
18145	852,800	116,700	6

a Vendor’s catalog No. [18].

**Table 3:** Apparent peak molecular weights *M*_GPC_ inferred from peak position on the chromatogram and the column calibration curve for linear polystyrene, and peak molecular weights *M*_LS_ obtained with the light scattering detector, for linear and star-branched polystyrenes. Also shown are the ratios *M*_LS_/*M*_GPC_

Sample No.	Elution volume at peak, units of 1.6/60 mL	*M*_GPC_ g/mol	*M*_LS_ g/mol	*M*_LS_/*M*_GPC_
Linear polystyrene				

705	1106	3×10^5^	4×10^5^	1.3
706	1053	5×10^5^	4×10^5^	0.8
1478	1287	3×10^4^	3×10^4^	1.0
1479	927	3×10^6^	3×10^6^	1.0

Star-branched polystyrene				

18143	1252	4×10^4^	5×10^5^	12
	1426	5×10^3^		
18144	1040	6×10^5^	8×10^5^	1.3
	1210	7×10^4^	1.4×10^5^	2.0
18145	981	1.3×10^6^	2×10^6^	1.5
	1142	1.7×10^5^		

## Footnotes

1 Certain commercial equipment, instruments, or materials are identified in this paper to specify adequately the experimental procedure. Such identification does not imply recommendation or endorsement by the National Institute of Standards and Technology, nor does it imply that the materials or equipment identified are necessarily the best available for the purpose.
